# Cost-effectiveness of sugemalimab plus chemotherapy as first-line therapy in advanced gastric cancer and gastroesophageal junction cancer.

**DOI:** 10.1080/07853890.2025.2583551

**Published:** 2025-12-09

**Authors:** Jiaming Zhu, Zhengxiong Li, Qiaoping Xu

**Affiliations:** ^a^ Fourth Clinical Medical College, Zhejiang Chinese Medical University, Affiliated Hangzhou First People’s Hospital, Hangzhou, P.R. China; ^b^ Department of Clinical Pharmacology, Key Laboratory of Clinical Cancer Pharmacology and Toxicology Research of Zhejiang Province, Cancer Center, Affiliated Hangzhou First People’s Hospital, Westlake University School of Medicine, Hangzhou, P.R. China; ^c^ School of Medical Informatics and Engineering, Xuzhou Medical University, Xuzhou, P.R. China

**Keywords:** Cost-effectiveness, sugemalimab, gastric cancer, chemoimmunotherapy, patient assistance programs

## Abstract

**Background:**

The GEMSTONE-303 trial demonstrated that sugemalimab combined with capecitabine and oxaliplatin (CAPOX) improved survival benefit in patients with advanced gastric/gastroesophageal junction cancer (GC/GEJC) and a programmed death-ligand 1 (PD-L1) combined positive score (CPS) ≥5. However, its cost-effectiveness in China remains unassessed.

**Methods:**

A partitioned survival model was developed from the Chinese healthcare payer perspective using data from the GEMSTONE-303 trial. Costs, quality-adjusted life-years (QALYs), and incremental cost-effectiveness ratios (ICERs) were evaluated over a 10-year horizon. Sensitivity analyses assessed model uncertainty, while subgroup and scenario analyses examined the impact of PD-L1 expression, drug pricing, and patient assistance programs (PAPs).

**Results:**

In the PD-L1 CPS ≥ 5 group, sugemalimab plus CAPOX yielded an incremental cost of $44,455.46 and 0.22 additional QALYs compared to chemotherapy, resulting in an ICER of $206,024.50/QALY. For patients with CPS ≥10, the ICER dropped to $173,182.90/QALY, but still exceeded the willingness-to-pay (WTP) threshold. One-way sensitivity analysis revealed sugemalimab price as the most influential parameter, requiring a 75.6% price reduction to achieve cost-effectiveness. A PAP reduced ICERs to $44,926.00/QALY and $39,513.96/QALY in the CPS ≥5 and CPS ≥10 subgroups, respectively.

**Conclusion:**

At current prices, sugemalimab plus CAPOX is not cost-effective as a first-line treatment for advanced GC/GEJC in China. It is essential to adopt a combination of targeted patient selection, price negotiation, and broader PAP access to bring the ICER below the WTP threshold. These findings inform reimbursement negotiations and highlight the need for stratified pricing strategies to optimize accessibility in economically diverse populations.

## 1. Introduction

As a leading oncological disease worldwide in the19th–20th centuries, gastric cancer (GC) has experienced substantial decreases in epidemiological burden following the establishment of early screening methodologies in contemporary medical practice. Nevertheless, GC remains the fifth most common malignant tumor globally. In certain regions, particularly in Asia, it continues to exhibit high incidence rates due to factors such as dietary habits [1]. The global macroeconomic cost of GC was estimated at $1.449 trillion (2017 data), accounting for 6.4% of the total economic burden of cancer worldwide, ranking sixth among all cancers. The disease burden may be even higher in middle-income countries [2].

Fortunately, numerous targeted agents for advanced GC are under extensive investigation, primarily targeting human epidermal growth factor receptor 2 (HER2), microsatellite instability-high/deficient mismatch repair, claudin 18.2 (CLDN18.2), vascular endothelial growth factor/vascular endothelial growth factor receptor, and fibroblast growth factor receptor (FGFR2), among others [3]. However, the consistent efficacy and broad applicability of these drugs still require improvement. For instance, trastuzumab, as the first approved targeted therapy for HER2+ patients, often fails due to HER2 loss [4]. Zolbetuximab and bemarituzumab demonstrate significant benefits only in patients with high CLDN18.2 and FGFR2b expression, respectively [3], necessitating rigorous immunohistochemical screening and continuous HER2 status monitoring. These requirements not only limit the eligible patient population but also increase the economic burden on cancer patients. Furthermore, programmed death-ligand 1 (PD-L1) targeted therapies have shown suboptimal outcomes. The KEYNOTE-062 trial revealed that pembrolizumab significantly benefits patients with PD-L1 combined positive score (CPS) ≥10 or MSI-H/dMMR tumor mutations [5], but fails to demonstrate survival advantages in patients with lower CPS scores [6]. Additionally, nivolumab, another PD-L1 inhibitor, exhibits differential efficacy between Asian populations and global data [7,8], with its therapeutic value in Asian cohorts remaining incompletely characterized.

PD-L1 (also known as CD274 or B7-H1) is a membrane receptor molecule expressed on tumor cells and target cells. Its expression is regulated by multiple factors, including mitogen-activated protein kinase or phosphatidylinositol 3-kinase-Akt pathway activation, Epstein-Barr virus infection, and inflammatory cytokines [9]. PD-1, the receptor for PD-L1, is predominantly expressed on CD8+ T cells and functions as an immune checkpoint, suppressing cytotoxic T cell activity and contributing to an immunologically “cold” tumor microenvironment. In 2018, Chen et al. discovered that tumor cells can release PD-L1-bearing exosomes, which bind to and inhibit CD8+ T cell function [10]. Consequently, sugemalimab, a monoclonal antibody specifically targeting PD-L1—blocks this immunosuppressive interaction, thereby “heating” the tumor immune microenvironment and exerting anti-cancer effects.

Notably, PD-L1 is not exclusively expressed on tumor cells; under physiological conditions, stromal cells also express PD-L1 to modulate immune response intensity. Thus, the CPS serves as a valuable tool to predict immunotherapy efficacy, with higher CPS scores generally indicating greater patient suitability for immune checkpoint blockade. Reflecting clinical practice, CPS thresholds guide treatment decisions, for instance, the Chinese Society of Clinical Oncology (CSCO) 2024 guidelines recommend pembrolizumab as a Category 1 A treatment for patients with CPS ≥ 10, while tislelizumab or nivolumab monotherapy is recommended as Category 1 A for those with CPS ≥ 5 [11].

Sugemalimab is a fully human, full-length PD-L1-targeting monoclonal antibody developed by CStone Pharmaceuticals. Its fully humanized structure confers low immunogenicity, minimizing the risk of anti-drug antibody formation and subsequent efficacy reduction. Notably, sugemalimab retains a functional Fc domain that enables antibody-dependent cell-mediated cytotoxicity, thereby enhancing tumor cell elimination. Through Fc engineering modifications, the antibody exhibits reduced activation of specific immune effector cells, protecting normal PD-L1-expressing tissues and further improving its safety profile. Since its initial approval by China’s National Medical Products Administration (NMPA) in 2021 for non-small cell lung cancer (NSCLC) treatment, sugemalimab has gained additional indications for relapsed/refractory extranodal NK/T-cell lymphoma (R/R ENKTL) and esophageal squamous cell carcinoma. In 2024, it received regulatory approvals in the European Union and United Kingdom for first-line treatment of metastatic non-small cell lung cancer, along with U.S. FDA clearance for clinical trials in R/R ENKTL [12].

The GEMSTONE-303 trial, the first Phase III study in China evaluating PD-L1 immunotherapy for advanced GC [13], prospectively enrolled 479 eligible cases meeting the criteria of PD-L1 CPS ≥ 5 in unresectable, locally advanced, or metastatic GC/GEJC, with the primary objective being efficacy assessment of sugemalimab plus capecitabine and oxaliplatin (CAPOX). The incorporation of sugemalimab demonstrated significant survival benefits, achieving a median overall survival of 15.64 months versus 12.65 months in the control cohort (hazard ratio [HR] 0.75; *p* = 0.006), representing a 3-month improvement. Concurrently, progression-free survival was prolonged to 7.62 months compared with 6.08 months (HR 0.66; *p* < 0.0001), corresponding to a 1.6-month advantage. The lower overall survival (OS) HR indicated significant mortality risk reduction with sugemalimab. CPS-stratified analysis revealed enhanced objective response rates in the CPS ≥ 10 subgroup compared to the CPS ≥ 5 population (71.4% vs. 68.6%), validating the preferential suitability of higher CPS thresholds for sugemalimab while confirming clinically meaningful benefits in moderate-CPS patients. Cross-trial comparison with the CheckMate-649 CPS ≥ 5 cohort showed concordant conclusions, with sugemalimab achieving comparable OS and progression-free survival (PFS) benefits to nivolumab despite shorter follow-up, thereby supporting its therapeutic superiority [8]. Safety analyses indicated no significant increase in severe adverse events (SAEs) with sugemalimab versus chemotherapy. Treatment-related adverse events (AEs) occurred in one-third of patients, predominantly platelet count reduction, contrasting favorably with the 59% SAE rate reported for nivolumab in CheckMate-649. This safety advantage further reinforces sugemalimab’s clinical profile. In 2024, the NMPA approved sugemalimab for advanced CPS ≥ 5 gastric adenocarcinomas, marking it as the first globally authorized PD-L1-targeted agent for GC. The CSCO subsequently classified it as a Category 1 A recommended first-line therapy.

The GEMSTONE-303 study demonstrated that sugemalimab plus CAPOX chemotherapy significantly improved both PFS and OS compared to placebo plus CAPOX in patients with unresectable locally advanced or metastatic GC/GEJC PD-L1 CPS ≥ 5, establishing it as a new standard of care for first-line GC therapy. Nevertheless, the high cost of immune checkpoint inhibitors poses substantial financial toxicity risks to certain patient populations, potentially widening existing healthcare disparities in GC management. Although cost-effectiveness analyses of sugemalimab in lung and esophageal cancers have been extensively reported, robust pharmacoeconomic evidence for its application in GC remains lacking. To address this gap, we conducted a cost-effectiveness evaluation of sugemalimab as a first-line treatment for unresectable, locally advanced, or metastatic GC/GEJC from the Chinese healthcare payer perspective. This study aimed to inform dynamic updates to national reimbursement policies, facilitate the design of tiered payment strategies, mitigate catastrophic health expenditure burdens at the individual level, and promote equitable access to this therapeutic advance across socioeconomic strata.

## 2. Methods

Implementation of the Consolidated Health Economic Evaluation Reporting Standards (CHEERS) 2022 guidelines governed this investigation’s reporting quality. Item-by-item validation against the 28-criterion checklist is systematically presented in Additional Material Table S1.

### 2.1. Therapeutic approaches and population

The study targeted patients aged 63 years with unresectable, locally advanced, or metastatic GC or GEJC, all immunohistochemically confirmed as HER2-negative. Key exclusion criteria included any prior systemic therapy for advanced or metastatic disease, any previous treatment with anti-PD-1/PD-L1 checkpoint inhibitors, a history of active or high-risk autoimmune disease, and the presence of unstable central nervous system metastases. Patients who had received prior adjuvant or neoadjuvant chemotherapy were eligible if disease progression occurred at least 6 months after the last dose, with this patient profile aligning with the baseline characteristics of the GEMSTONE-303 trial. This study evaluated the cost-effectiveness of sugemalimab plus CAPOX versus placebo plus CAPOX. Notably, all enrolled patients underwent centralized PD-L1 assessment (Ventana SP263 antibody) demonstrating a CPS ≥ 5. Patients were stratified by CPS score (CPS ≥ 5, CPS ≥ 10) and randomized 1:1 to either the sugemalimab treatment group or placebo group. Patients received either sugemalimab or placebo 1200 mg intravenously every 21 days for up to 2 years. Both groups received CAPOX chemotherapy (oral capecitabine 1000 mg/m^2^ twice daily on days 1–14 plus intravenous oxaliplatin 130 mg/m^2^ on day 1), with treatment cycles repeated every 21 days for up to 6 cycles. Disease progression or intolerable AEs as clinical trial protocol prescribed were defined as treatment discontinuation criteria [13]. Additionally, the clinical trial reported post-progression anti-tumor drug use: 17.8% in the sugemalimab group and 23.5% in the placebo group received other immune checkpoint inhibitors; 49.4% and 57.9% received chemotherapy; while 24.9% and 23.5% received other treatments. The study referenced both the 2024 CSCO Gastric Cancer Guidelines and the NCCN Clinical Practice Guidelines for Gastric Cancer [14]. For modeling purposes, it was assumed that second-line immune checkpoint inhibitor therapy for the CPS ≥ 5 subgroup would randomly use either nivolumab or pembrolizumab PD-1 inhibitors, while the CPS ≥ 10 subgroup would specifically receive pembrolizumab. Second-line chemotherapy used either docetaxel or irinotecan, and other treatments included ramucirumab or best supportive care [13]

### 2.2. Model structure

**Figure 1.**
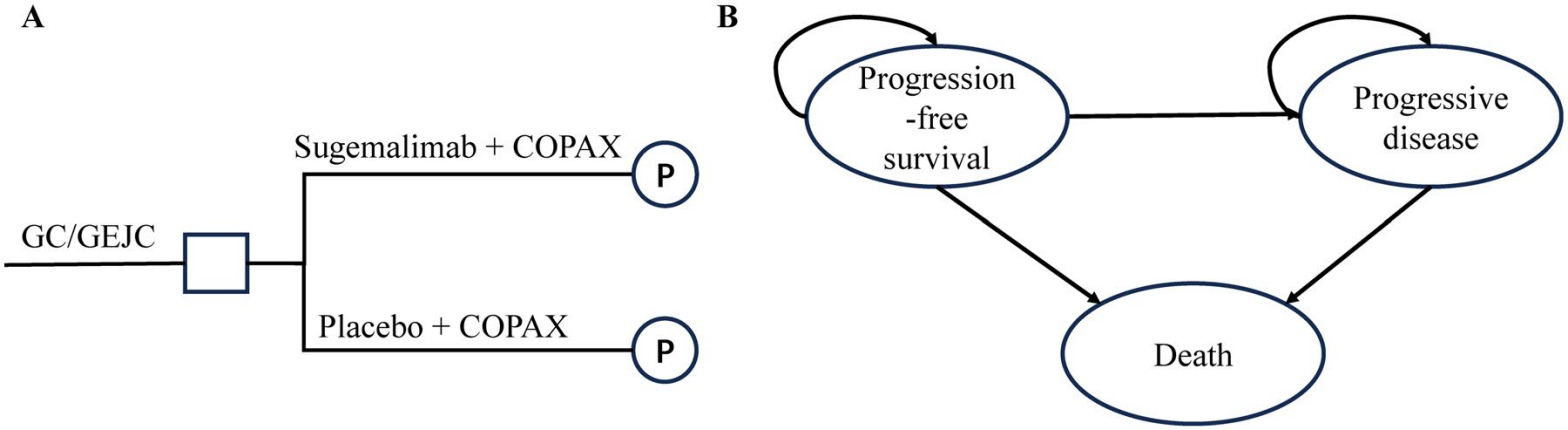
The structure of the decision tree (A) and the partitioned survival model(B). Abbreviations: GC/GEJC, Gastric/gastroesophageal junction cancer; CAPOX, capecitabine and oxaliplatin.

This study constructed a partitioned survival model using TreeAge Pro 2022 software (TreeAge, Williamstown, MA), which simulated disease progression by dividing the patient cohort into mutually exclusive health states: PFS, progressed disease (PD), and death (Figure 1). State occupancy probabilities were calculated through parametric curve decomposition, where proportion of PFS was directly to the integral under the PFS curve, while the PD proportion determined by the area difference between OS and PFS curves. Deaths were calculated as 1 minus the combined proportions of patients in PFS and PD states. Patients could only transition from progression-free to progressed state without therapeutic reversal, and the death state was absorbing with no possibility of transitioning to other states. Given the poor prognosis of advanced GC/GEJC, with a 5-year survival rate of approximately 5% [15], a 10-year time horizon was adopted in the base-case analysis. The simulated cohort entered the cycle in the PFS state, with a cycle length of 21 days, consistent with the sugemalimab dosing interval in the clinical trial. According to the China Guidelines for Pharmacoeconomic Evaluations (2020) [16], the willingness-to-pay (WTP) threshold was set at 3 times China’s 2024 per capita GDP ($40,334 per quality-adjusted life year [QALY]). Simulation results generated total costs, QALYs, and the ICER for comparative analysis, a 5% annual discount rate was applied to convert future costs and utilities to present values. If the ICER was below the WTP threshold, adding sugemalimab to chemotherapy would be considered cost-effective; otherwise, the sugemalimab-chemotherapy combination would not be economically recommended for advanced GC patients under current conditions (Figure 1).

### 2.3. Clinical data

Since the original individual patient data (IPD) were not available, this study first extracted PFS time and OS survival data from the reported Kaplan-Meier (KM) survival curves using the GetData Graph Digitizer platform (version 2.26). Then, the R software (version 4.3.2) was used to reconstruct IPD (Figure S1 in Additional Material). Based on Guyot et al.’s study [17], five distributions including Exponential, Weibull, Gompertz, Log-logistic and Log-normal were selected to fit the reconstructed IPD using maximum likelihood estimation (MLE) enabling probabilistic prediction of post-trial survival trajectories. The optimal distribution was finally determined through visual inspection and information criterion values. The optimal parametric distribution was selected by minimizing both the Akaike Information Criterion (AIC) and Bayesian Information Criterion (BIC), combined with visual confirmation of concordance between the fitted KM curves and the original survival data (Additional Material Table S2 and Figure S2). The Log-logistic distribution was ultimately determined to be the optimal distribution for extrapolating both OS and PFS curves (Table 1).

**Table 1.** Summary of survival model parameters fitted to Kaplan-Meier curves.

Group	Model	Parameters
CPS ≥ 5	Log-logistic survival model of OS of sugemalimab PD-L1 CPS ≥ 5	shape = 1.865; scale = 15.448
	Log-logistic survival model of OS of placebo PD-L1 CPS ≥ 5	shape = 1.835; scale = 12.358
	Log-logistic survival model of PFS of sugemalimab PD-L1 CPS ≥ 5	shape = 1.933; scale = 7.691
	Log-logistic survival model of PFS of placebo PD-L1 CPS ≥ 5	shape = 2.125; scale = 5.613
CPS ≥ 10	Log-logistic survival model of OS of sugemalimab PD-L1 CPS ≥ 10	shape = 1.857; scale = 16.879
	Log-logistic survival model of OS of placebo PD-L1 CPS ≥ 10	shape = 1.654; scale = 11.917
	Log-normal survival model of PFS of sugemalimab PD-L1 CPS ≥ 10	meanlog = 2.1228; sdlog = 0.849
	Log-normal survival model of PFS of placebo PD-L1 CPS ≥ 10	meanlog = 1.6621; sdlog = 0.8727

Abbreviations: OS, overall survival; PFS, progression-free survival; CPS, combined positive score; PD-L1, programmed death-ligand 1.

### 2.4. Cost and utility

This study conducted a cost-effectiveness analysis from the perspective of Chinese healthcare payers, exclusively incorporating direct medical costs encompassing expenses of genetic testing, first-line therapeutic agents, second-line regimen costs, drug administration costs, follow-up examination costs, end-of-life care costs, and management costs for SAEs (Grade ≥ 3) with an incidence greater than 5% in the sugemalimab group, which were assumed to be accounted for only in the first cycle of the model. The specific incidence percentages for these SAEs were sourced from the GEMSTONE-303 trial and are detailed in Table 2. The dosage and costs of chemotherapy drugs were based on an adult patient with a weight of 65 kg and a body surface area of 1.72 m^2^ [27]. Drug costs were partially derived from the median 2024 bid-winning prices in the www.yaozh.com database [28]. To assess the impact of price variability, the lowest bid-winning price was applied as the lower bound in the sensitivity analysis, while the upper bound was calculated by adding the price difference between the base-case price and the lower-bound price to the base-case estimate. This approach allows us to explore the potential impact of price fluctuations within the context of centralized procurement in Chinese public hospitals [29,30]. To reflect real-world clinical practice, vial sharing between patients was not permitted in our model. Drug wastage was explicitly considered, assuming that each opened single-use vial was billed in full according to its labeled price, regardless of the actual amount administered. Follow-up examination items included those explicitly listed in the clinical study and additional tests recommended in the 2024 CSCO Gastric Cancer Diagnosis and Treatment Guidelines for advanced GC, as advised by consulting relevant experts. The pricing for these examinations was sourced from ybj.zj.gov.cn and published references. The management costs of SAEs were based on published literature [18,21]. To minimize price fluctuations caused by differences in data sources and inflation, all costs were adjusted to 2024 USD using China’s historical Consumer Price Index (CPI) data and the formula provided by www.inflationtool.com, with an exchange rate of ($1 = CNY 7.1217 (2024 average exchange rate)) [31].

**Table 2.** Model input parameters.

Parameters	Baseline	SA range	Distribution	References
**Cost of drug**				
Sugemalimab per 600 mg	1,737.65	1,390.12-2,085.18	Gamma	yaozh.com
Capecitabine per 500 mg	0.26	0.25-0.27	Gamma	yaozh.com
Oxaliplatine per 50 mg	17.45	4.26-30.64	Gamma	yaozh.com
Drug injection per time	2.99	2.39-3.59	Gamma	[18]
**Cost of second-line treatment per cycle**				
Nivolumab	3,799.12	3,039.30–4,558.94	Gamma	yaozh.com
Pembrolizumab	5,031.94	4,025.56–6,038.33	Gamma	yaozh.com
Docetaxel	36.23	2.90–69.56	Gamma	yaozh.com
Irinotecan	749.30	75.68–1422.92	Gamma	yaozh.com
Ramucirumab	3,285.52	2,628.42–3,942.63	Gamma	yaozh.com
Paclitaxel	198.67	46.00–351.34	Gamma	yaozh.com
**Proportion of second-line therapy in sugemalimab group (%)**				
Nivolumab	8.9	7.12–10.68	Beta	–
Pembrolizumab	8.9	7.12–10.68	Beta	–
Docetaxel	16.5	13.20–19.80	Beta	–
Irinotecan	16.5	13.20–19.80	Beta	–
Ramucirumab	24.9	19.92–29.88	Beta	–
Paclitaxel	16.5	13.20–19.80	Beta	–
BSC	46.5	37.20–55.80	Beta	–
**Proportion of second-line therapy in placebo group (%)**				
Nivolumab	11.8	9.44–14.16	Beta	–
Pembrolizumab	11.8	9.44–14.16	Beta	–
Docetaxel	19.3	15.44–23.16	Beta	–
Irinotecan	19.3	15.44–23.16	Beta	–
Ramucirumab	23.5	18.80–28.20	Beta	–
Paclitaxel	19.3	15.44–23.16	Beta	–
BSC	36.1	28.88–43.32	Beta	–
**Cost of monitoring per time**				
Gastroscope	53.72	42.97–64.46	Gamma	[19]
Enhanced CT	10.30	8.24–12.36	Gamma	[20]
Laboratory examination	109.75	87.80–131.71	Gamma	[20]
Helicobacter pylori test	16.85	13.48–20.22	Gamma	[20]
Nutritional tests (Fe^2+^, Vitamin B_12_)	6.46	5.17–7.75	Gamma	[20]
HER2 gene test	168.90	134.80–202.20	Gamma	Local estimate
PD-L1 expression levels test	168.90	134.80–202.20	Gamma	Local estimate
Cost of BSC per cycle	127.66	102.13–153.19	Gamma	[21]
Cost of terminal care	1,469.00	1,175.20–1,762.80	Gamma	[21]
Cost of follow up	79.02	63.21–94.82	Gamma	[22]
**Probability of SAEs (grade ≥ 3) in sugemalimab group (%)**				
Platelet count decreased	18.3	14.64–21.96	Beta	[13]
Neutrophil count decreased	14.1	11.28–16.92	Beta	[13]
Anemia	10.8	8.64–12.96	Beta	[13]
White blood cell counts decreased	6.6	5.28–7.92	Beta	[13]
**Probability of SAEs (grade ≥ 3) in placebo group (%)**				
Platelet count decreased	16.0	12.80–19.20	Beta	[13]
Neutrophil count decreased	14.3	11.44–17.16	Beta	[13]
Anemia	7.2	5.76–8.64	Beta	[13]
White blood cell counts decreased	3.0	2.40–3.60	Beta	[13]
**Cost of managing SAEs (grade ≥ 3) per event**				
Platelet count decreased	1,614.24	1,291.39–1,937.09	Gamma	[21]
Neutrophil count decreased	123.27	98.62–147.92	Gamma	[21]
Anemia	148.72	118.98–178.47	Gamma	[21]
White blood cell counts decreased	501.64	401.31–601.97	Gamma	[18]
Discount rate	0.05	0–0.08	Fixed	[23]
Body surface (m^2^)	1.72	1.38–2.06	Gamma	[24]
Weight (kg)	65.00	52.00–78.00	Gamma	[25]
**Health state utilities**				
Progressive disease	0.577	0.46–0.69	Beta	[26]
Progression-free survival	0.797	0.64–0.96	Beta	[26]
**Disutility**				
Platelet count decreased	0.090	0.07–0.11	Beta	[25]
Neutrophil count decreased	0.150	0.12–0.18	Beta	[25]
Anemia	0.073	0.06–0.09	Beta	[25]
White blood cell counts decreased	0.200	0.16–0.24	Beta	[18]

Abbreviations: SA, sensitive analysis; BSC, best supportive care; CT, computerized tomography; SAEs, severe adverse events; HER2, human epidermal growth factor receptor 2.

Utility values quantified the impact of interventions on patient health outcomes, typically represented as a validated scale ranging from 0 (equivalent to death) to 1 (optimal health state). In this study, the utility values for PFS and PD states were referenced from published literature [26]. The occurrence of SAEs may reduce patients’ quality of life, and this reduction was quantified using disutility values (Table 2).

### 2.5. Subgroup analysis

Given the theoretical basis that higher CPS values may significantly enhance the therapeutic efficacy of PD-L1 inhibitors, we evaluated cost-effectiveness in patients with PD-L1 CPS ≥ 10 by reconstructing IPD through fitting and extrapolation methods, utilizing OS and PFS curve data from the PD-L1 CPS ≥ 10 subgroup in clinical trials.

### 2.6. Sensitivity analyses

To assess the impact of model variables on final outcomes, this study employed one-way sensitivity analysis (OWSA) by varying each parameter from 80% to 120% of its baseline value excluding drug costs, with the discount rate specifically ranging from 0 to 0.08. The top 20 variables significantly influencing the ICER were visualized using a tornado diagram. Additionally, probabilistic sensitivity analysis (PSA) incorporated 5,000 Monte Carlo simulations through repeated random sampling to comprehensively evaluate parameter uncertainty and validate model robustness. Costs, body surface area, and body weight were assumed to follow Gamma distributions, while utility values, incidence of SAEs, and probabilities of receiving second-line treatment were modeled with Beta distributions. The results were visualized through ICER scatter plots and cost-effectiveness acceptability curves (CEACs).

### 2.7. Scenario analyses

Several scenario analyses were conducted to evaluate the robustness of the model under different assumptions. We first assessed the impact of sugemalimab price reductions (25%, 50%, and 75%) on total costs and cost-effectiveness. In addition, we incorporated Pfizer’s Patient Assistance Program (PAP), which was implemented in three phases: Phase 1 offers two additional free treatment cycles after purchasing two cycles, while subsequent phases provide 25 and 3 free cycles for purchasing two and one treatment cycles, respectively. Considering the real-world barriers to PAP coverage, such as administrative hurdles and regional variations, we explored a scenario in which only 50% of patients receive PAP. The cost-effectiveness of PAP was evaluated separately for patients with CPS ≥ 5 and CPS ≥ 10.

Furthermore, we considered a scenario where drug acquisition costs did not account for any leftover drug wastage. To examine the effect of the simulation time horizon, we also used extended time frames, including 15-year and lifetime horizons. We evaluated the impact of different distribution choices by running models with various parametric distributions for PFS and OS. Finally, to reflect regional disparities, all scenarios were assessed against the WTP threshold for high-income regions, such as Beijing WTP=$96,057.6/QALY, to determine their influence on cost-effectiveness acceptability probabilities.

## 3. Results

### 3.1. Base-case analysis and subgroup analysis

The base-case analysis demonstrated that for patients with PD-L1 CPS ≥ 5, the annual total cost of the sugemalimab plus CAPOX chemotherapy regimen was $80,259.68. Compared to the standard chemotherapy group $35,804.22, the sugemalimab combination was associated with an incremental cost of $44,455.46 for a gain of 0.22 QALYs, yielding an ICER of $206,024.50/QALY. This exceeded the WTP threshold of $40,334/QALY, indicating a lack of cost-effectiveness. In the PD-L1 CPS ≥ 10 subgroup, the sugemalimab plus CAPOX regimen incurred a total cost of $89,263.59, with an incremental cost of $48,508.72 versus the placebo group. The intervention provided an additional 0.28 QALYs compared to placebo plus CAPOX, resulting in an ICER of $173,182.90/QALY. These findings demonstrate that sugemalimab plus CAPOX failed to show cost-effectiveness compared to chemotherapy alone in China, both in populations with CPS ≥ 5 and CPS ≥ 10 (Table 3).

**Table 3.** Result of base-case analysis.

Group	Strategy	Total cost ($)	Incremental Cost ($)	QALYs	Incremental QALYs	ICER ($/QALY)
PD-L1 CPS ≥ 5	Sugemalimab	80,259.68	44,455.46	1.15	0.22	206,024.50
	Placebo	35,804.22	–	0.93	–	–
PD-L1 CPS ≥ 10	Sugemalimab	89,263.59	48,508.72	1.23	0.28	173,182.90
	Placebo	40,750.02	–	0.95	–	–

Abbreviations: CPS, combined positive score; QALYs, quality adjusted life years.

### 3.2. One way sensitivity analysis

**Figure 2.**
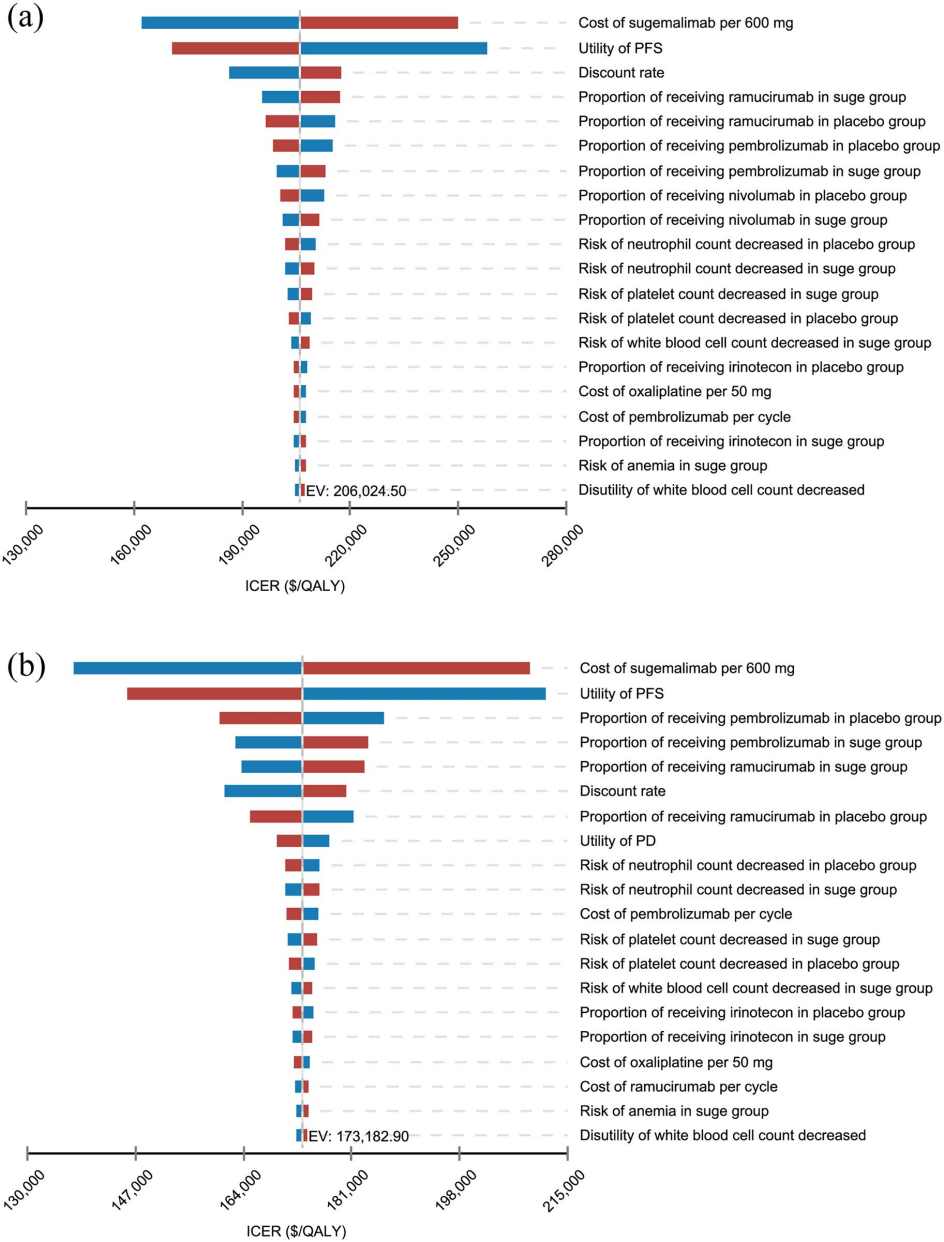
Tornado diagram of one-way sensitivity analysis in PD-L1 CPS ≥ 5(A) and PD-L1 CPS ≥ 10 (B). Abbreviations: PFS, progression-free survival; PD, progressed disease; QALY, quality adjusted life years; ICER, incremental cost-effectiveness ratios; EV, expected value; suge, sugemalimab.

The OWSA showed that for both the PD-L1 CPS ≥ 5 and CPS ≥ 10 patient subgroups, the top two factors most significantly influencing the ICER were the unit price of sugemalimab and the utility values for PFS. This indicated that ±20% price fluctuations in sugemalimab substantially affected the ICER, but did not reduce it below the WTP threshold of $40,334/QALY. Variations of ±20% in other factors did not reverse the conclusion that sugemalimab plus CAPOX chemotherapy lacked cost-effectiveness (Figure 2).

### 3.3. Probabilistic sensitivity analysis

**Figure 3.**
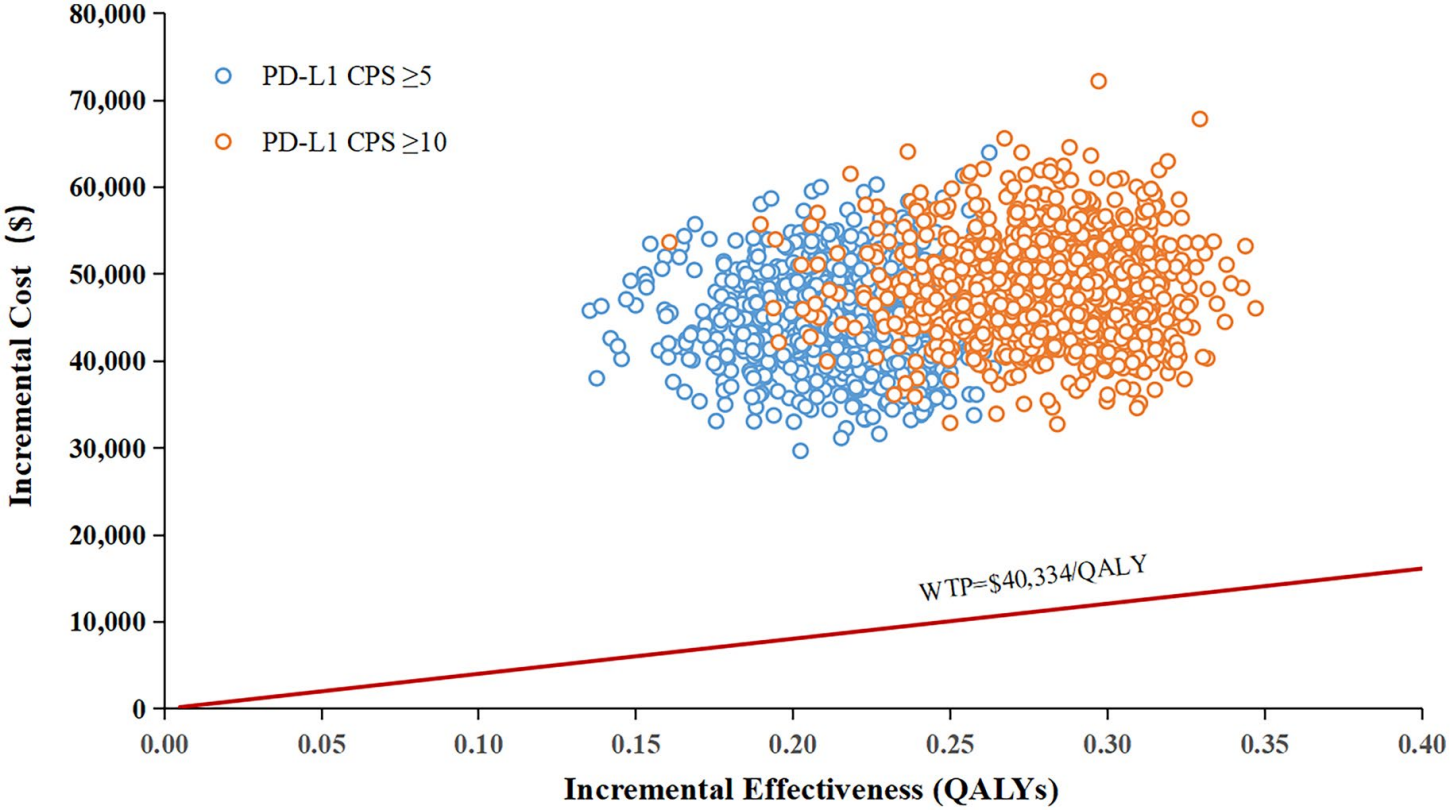
The incremental cost-effectiveness scatter plot of Sugemalimab plus CAPOX compared to placebo plus CAPOX in PD-L1 CPS ≥ 5 and CPS ≥ 10 subgroup. Abbreviations: QALY, quality adjusted life years; WTP, willingness-to-pay; PD-L1, programmed death-ligand 1; CPS, combined positive score.

**Figure 4.**
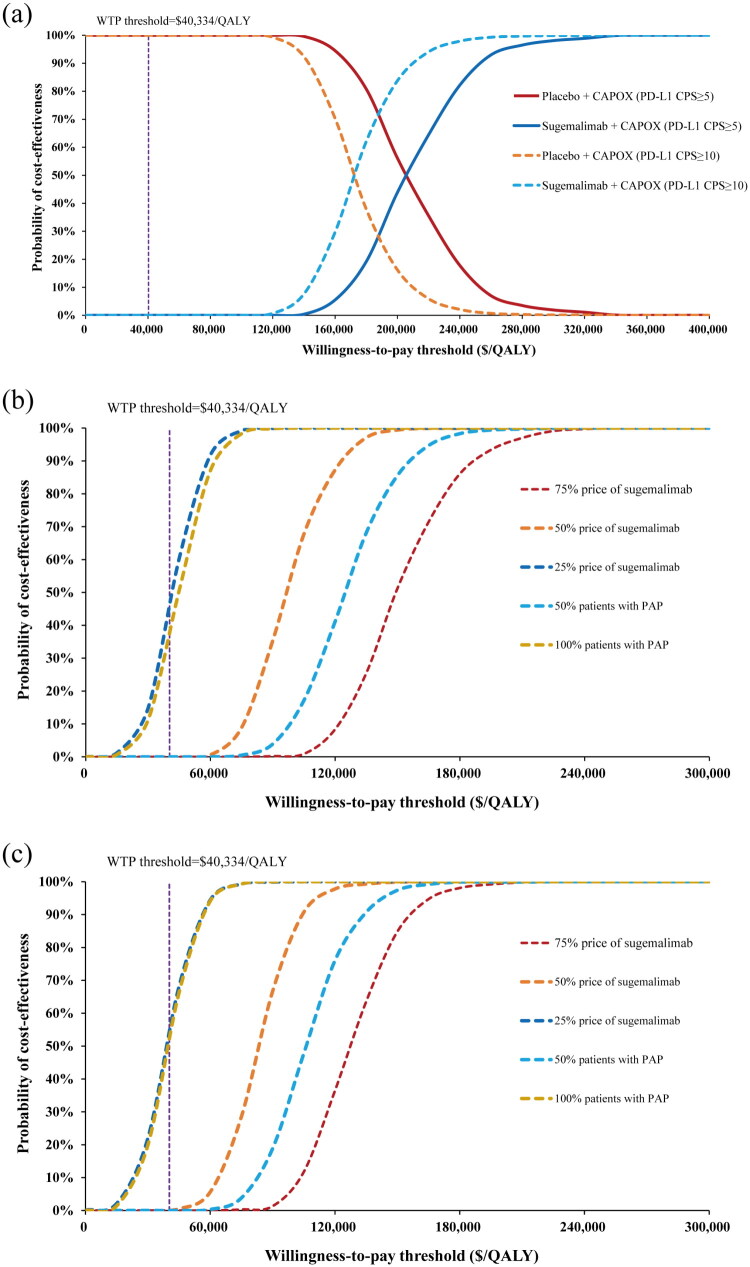
Cost-effectiveness acceptability curves for sugemalimab plus CAPOX: (a) in PD-L1 CPS ≥ 5 and CPS ≥10 subgroups; (b) under different scenario analyses for patients with PD-L1 CPS ≥ 5; and (c) under different scenario analyses for patients with PD-L1 CPS ≥10. Abbreviations: QALY, quality adjusted life years; WTP, willingness-to-pay; PAP, patient assistance program CPS, combined positive score; PD-L1, programmed death-ligand 1.

The PSA scatter plots demonstrated that all data points for both CPS ≥ 5 and CPS ≥ 10 subgroups remained above the WTP threshold line, indicating a 0% acceptability probability for sugemalimab plus CAPOX versus CAPOX chemotherapy at a WTP of $40,334/QALY (Figure 3). The CEACs analysis demonstrated a 0% likelihood of sugemalimab-based regimen meeting cost-effectiveness criteria at the prespecified threshold, with consistent findings across all CPS subgroups. When the WTP approached $210,000/QALY and $170,000/QALY, the cost-effectiveness probabilities reached 50% for the CPS ≥ 5 cohort and CPS ≥ 10 subgroup, respectively (Figure 4a).

### 3.4. Scenario analyses

Simulated price reductions for sugemalimab revealed that in CPS ≥ 5 group when the drug price was reduced by 75% the total cost of sugemalimab plus CAPOX chemotherapy decreased to $44,782.22, yielding an ICER of $41,608.06/QALY, with a cost-effectiveness acceptability probability of 47.5%. Further analysis identified that a 75.6% price reduction, equating to $477.85 per 600 mg of sugemalimab, produced an ICER below the WTP threshold. For high-income regions like Beijing, a 50% price reduction resulted in a cost-effectiveness acceptability probability of 52.5%. For the CPS ≥ 10 subgroup, a 75% price reduction showed cost-effectiveness in China, while a 43% price reduction showed cost-effectiveness in Beijing. Among CPS ≥ 5 and CPS ≥ 10 subgroup patients enrolled in the PAP, the sugemalimab group incurred an ICER of $44,926.00/QALY and $39,513.96/QALY, respectively. When the proportion of patients receiving PAP was reduced to 50%, the ICER increases to $125,475.24/QALY and $106,384.43/QALY, respectively. Extending the simulation time horizon and excluding drug wastage had little impact on the ICER (Table 4). The results of the distribution choice analysis were also robust, as shown in Supplementary Materials Table S3.

**Table 4.** Result of scenario analysis.

Scenario	Group	Total cost ($)	QALYs	ICER ($/QALY)	Probability of being cost-effective in China	Probability of being cost-effective in Beijing
PD-L1 CPS ≥ 5 75% price of sugemalimab	Sugemalimab plus CAPOX	68,433.80	1.15	151,219.00	0%	0.50%
PD-L1 CPS ≥ 5 50% price of sugemalimab	Sugemalimab plus CAPOX	56,608.01	1.15	96,413.53	0%	52.50%
PD-L1 CPS ≥ 5 25% price of sugemalimab	Sugemalimab plus CAPOX	44,782.22	1.15	41,608.06	47.50%	99.80%
PD-L1 CPS ≥ 10 75% price of sugemalimab	Sugemalimab plus CAPOX	76,691.89	1.23	128,304.67	0%	4.30%
PD-L1 CPS ≥ 10 50% price of sugemalimab	Sugemalimab plus CAPOX	64,120.19	1.23	83,426.44	0%	79.20%
PD-L1 CPS ≥ 10 25% price of sugemalimab	Sugemalimab plus CAPOX	51,548.49	1.23	38,548.21	54.50%	100%
PD-L1 CPS ≥ 5 with PAP	Sugemalimab plus CAPOX	45.498.16	1.15	44,926.00	36.70%	100%
PD-L1 CPS ≥ 10 with PAP	Sugemalimab plus CAPOX	51,819.03	1.23	39,513.96	51.70%	100%
PD-L1 CPS ≥ 5 with 50% PAP coverage	Sugemalimab plus CAPOX	62,878.87	1.15	125,475.24	0%	6.30%
PD-L1 CPS ≥ 10 with 50% PAP coverage	Sugemalimab plus CAPOX	70,541.31	1.23	106,348.43	0%	30.80%
PD-L1 CPS ≥ 5 (time horizon = 15 years)	Sugemalimab plus CAPOX	81,322.96	1.17	199,117.31	0%	0%
	Placebo plus CAPOX	36,776.72	0.95	–	–	–
PD-L1 CPS ≥ 5 (lifetime horizon)	Sugemalimab plus CAPOX	81,764.42	1.18	196,406.38	0%	0%
	Placebo plus CAPOX	37,193.36	0.96	–	–	–
PD-L1 CPS ≥ 5 excluding drug wastage	Sugemalimab plus CAPOX	80,167.20	1.15	206,484.37	0%	0%
	Placebo plus CAPOX	35,612.51	0.93	–	–	–

Abbreviations: QALYs, quality adjusted life years; CAPOX, capecitabine and oxaliplatin; PAP, patient assistance programs.

## 4. Discussion

The GEMSTONE-303 trial demonstrated that sugemalimab significantly improves survival outcomes in patients with advanced GC/GEJC and a CPS ≥ 5. This study represented the first cost-effectiveness analysis of sugemalimab combined with CAPOX chemotherapy for locally advanced or metastatic GC/GEJC from the perspective of Chinese healthcare payers. Base-case analysis demonstrated that although sugemalimab use increased patient QALYs by 0.22, benefiting quality of life, the combination of sugemalimab with CAPOX was not cost-effective for patients with CPS ≥ 5 due to its high drug price. Subgroup analysis revealed that for patients with CPS ≥ 10, while the ICER remained above the WTP threshold, the ICER was reduced compared with the CPS ≥ 5 population, indicating improved cost-effectiveness of this regimen in the CPS ≥ 10 subgroup. This finding was consistent with the GEMSTONE-303 trial, which showed that patients with CPS ≥ 10 had lower risks of mortality and disease progression than those with CPS ≥ 5. We also observed that the total cost for the CPS ≥ 10 subgroup was higher than that for the CPS ≥ 5 subgroup. Further exploration revealed that in the sugemalimab treatment group, although patients with higher CPS scores experienced longer PFS compared to those with intermediate CPS scores, their OS benefit was more pronounced. Consequently, the higher QALYs in the CPS ≥ 10 subgroup were primarily driven by the accumulation of time in the PD state. From a cost perspective, the higher costs in the CPS ≥ 10 subgroup were also mainly attributed to the extended time spent in the PD state. This suggested that the increased costs in the CPS ≥ 10 subgroup were primarily due to prolonged treatment durations, specifically the extended post-progression treatment period. Additionally, we observed that in the chemotherapy regimen, the total costs and OS for the CPS ≥ 10 subgroup were also higher than for the CPS ≥ 5 subgroup. Further analysis indicated that this was mainly due to the extended post-progression survival, which may be associated with the crossover use of PD-1 and PD-L1 inhibitors in the second-line chemotherapy setting, leading to better efficacy in the CPS ≥ 10 subgroup.

OWSA demonstrated that PFS utility values exerted significantly greater influence on the ICER than PD utility values, which was likely attributable to the greater time spent in the PFS state. Scenario analysis of price reduction revealed that a minimum 75.6% price reduction would be required to achieve cost-effectiveness for the sugemalimab plus CAPOX regimen. Currently, sugemalimab is undergoing negotiations for inclusion in the national medical insurance system. Compared to similar immune checkpoint inhibitors such as penpulimab and serplulimab, which experienced price reductions of over 70% upon reimbursement, this price reduction is deemed feasible in real-world settings. Moreover, in high-income regions like Beijing, China, cost-effectiveness substantially improved when sugemalimab was priced at 50% of its original cost, suggesting these regions may benefit earlier from its economic advantages.

Additionally, since the maximum treatment duration for sugemalimab is two years, extending the simulation time horizon improved cost-effectiveness. This is due to the accumulation of costs in the early phase of the combination therapy, while the long-term efficacy of immunotherapy allows incremental QALYs to continue accumulating after treatment discontinuation. Fortunately, for patients who meet the indication criteria for sugemalimab, the PAPs substantially reduced out-of-pocket financial burdens through medication support, lowering the ICER to $44,926/QALY, which is well approaching the WTP threshold. Unlike drug price reductions (a long-term trend with limited short-term feasibility), PAPs offer immediate economic relief for financially constrained patients requiring urgent treatment. In this context, prioritizing PAP implementation over price reductions proves critical for economically vulnerable populations, whereas price adjustments (while benefiting all patients requiring sugemalimab and potentially enhancing market penetration) may compromise manufacturer profitability. It is important to note that, although current policies stipulate that all patients meeting the eligibility criteria can access the PAP, in real-world settings, the implementation of PAPs may vary considerably across regions and institutions. Barriers such as limited hospital participation, complex administrative procedures, and insufficient awareness among patients or physicians can prevent PAP from covering all treatment cycles. In a scenario where 50% of patients receive PAP, the cost-effectiveness of sugemalimab in both the CPS ≥ 5 and CPS ≥ 10 subgroups remains suboptimal. These findings suggested that the impact of PAPs in real-world settings was largely dependent on their coverage and operational efficiency. Additionally, incorporating sugemalimab into risk-sharing agreements presents an alternative strategy to mitigate payment uncertainty for healthcare systems - such as manufacturer rebates for unmet efficacy outcomes - thereby distributing financial risks among patients, governments, and manufacturers through more equitable commercialization strategies.

Previous economic evaluations have assessed the cost-effectiveness of various PD-1/PD-L1 inhibitors in advanced GC/GEJC in China, revealing divergent conclusions. The results for nivolumab and pembrolizumab showed high ICERs of $278,658.71/QALY and $177,405.83/QALY, respectively, deeming them not cost-effective [32,33]. In stark contrast, analyses of sintilimab and tislelizumab demonstrated favorable economic profiles across all PD-L1 expression levels [18,34] (Table 5). Notably, with the exception of the analysis of nivolumab, where the time horizon was set to 5 years and the recruited population had a CPS ≥ 5, the other three studies used a 10-year time horizon and did not specify PD-L1 expression levels for patient eligibility. In terms of incremental QALYs, pembrolizumab achieved an increment of 0.29, outperforming sugemalimab, while the other immune checkpoint inhibitors yielded incremental QALYs that were comparable to those of sugemalimab. Regarding costs, nivolumab and pembrolizumab had costs exceeding $4,800 per treatment cycle, whereas sintilimab and tislelizumab, due to their inclusion in the National Reimbursement Drug List (NRDL), were priced below $700 per treatment cycle. Furthermore, studies have shown that when the price of nivolumab decreases by 90% [32], reducing the per-cycle treatment cost to approximately $500, it becomes cost-effective. This highlights that the drug price is the primary factor driving the ICER disparity between these therapies.

**Table 5.** Comparison of PD-1/PD-L1 inhibitors in economic evaluations.

Drug	Clinic trial	Contrast treatment	Total cost ($)	QALYs	ICER($/QALY)	Conclusion	Ref.
Nivolumab	CheckMate-649	Nivolumab plus chemotherapy VS placebo plus chemotherapy	81,190.00	1.11	278,658.71	Nivolumab is not cost-effective for gastric cancer patients with a PD-L1 CPS ≥ 5 in China, but a 90% price reduction would make it cost-effective.	[32]
Pembrolizumab	KEYNOTE-859	Pembrolizumab plus chemotherapy VS placebo plus chemotherapy	191,547.03	2.94	171,405.83	Pembrolizumab is not cost-effective for gastric cancer patients in China	[33]
Pembrolizumab	KEYNOTE-859	Pembrolizumab plus chemotherapy VS nivolumab plus chemotherapy	11,693.00	1.60	−331,696.00	Pembrolizumab is cost-effective in China but lacks cost-effectiveness in the US and the UK	[21]
Sintilimab	ORIENT-16	Sintilimab plus chemotherapy VS placebo plus chemotherapy	21,503.91	1.12	21,239.29	Sintilimab remains cost-effective across all PD-L1 CPS levels	[18]
Tislelizumab	RATIONALE-305	Tislelizumab plus chemotherapy VS placebo plus chemotherapy	21,297.65	1.00	37,768.48	Tislelizumab demonstrated comparable WTP and ICER in PD-L1-positive patients, while showing significantly better cost-effectiveness in unresectable GC/GEJC cancer with TAP ≥5%.	[34]

Abbreviations: QALYs, quality-adjusted life years; ICER, incremental cost-effectiveness ratio; CPS, combined positive score; GC/GEJC, gastric/gastroesophageal junction cancer; TAP, tumor area positivity score.

However, when comparing high-priced, non-reimbursed therapies, superior clinical efficacy can become the key determinant of the ICER. This was evident in the subgroup analysis of patients with PD-L1 CPS ≥ 10. Here, pembrolizumab achieved a significantly higher incremental QALYs gain than sugemalimab (0.67 vs. 0.28). Despite pembrolizumab’s higher treatment cost ($5,325 per cycle) compared to sugemalimab ($3,475 per cycle), its substantial efficacy advantage was sufficient to produce a more favorable ICER ($109,534.13/QALY) compared to that of sugemalimab in the same subgroup. These findings suggested that in the absence of major price negotiations, a remarkable improvement in clinical benefit is crucial for achieving better relative cost-effectiveness.

Immunohistochemical (IHC) PD-L1 biomarker assessment has been universally adopted as enrollment criteria or stratification factors in PD-1/PD-L1 inhibitor trials. However, current PD-L1 IHC assays like 22C3 and 28-8 exhibit substantial heterogeneity in scoring methodology. Notably, the 28-8 assay may result in higher PD-L1 CPS scores and positivity rates compared to other platforms [35], highlighting the lack of standardized CPS thresholds. This methodological variability is exemplified by the 22C3 antibody in KEYNOTE-859 versus SP263 in CheckMate-649/GEMSTONE-303, potentially contributing to discrepancies in economic evaluations. Helicobacter pylori infection influences GC immunotherapy through chronic inflammation-induced PD-1/PD-L1 pathway activation and “hot tumor microenvironment” remodeling [36]. Since PD-1/PD-L1 inhibitors mechanistically target this axis, enhanced efficacy is observed in H. pylori-positive patients. We recommend pretreatment H. pylori screening to stratify populations, which may improve therapeutic and economic assessment precision.

From a clinical trial perspective, this may introduce interference in cost-effectiveness analyses, while the study itself possesses a number of limitations. First, a primary source of uncertainty in our model stems from the long-term extrapolation of survival outcomes. The follow-up duration in the GEMSTONE-303 trial is finite, necessitating the use of parametric functions to project OS and PFS curves over a lifetime horizon. Although we selected the best-fitting statistical distribution based on established criteria validated the robustness of our findings through sensitivity analysis using alternative distributions, these long-term projections may not perfectly reflect the actual survival of patients, introducing a degree of uncertainty into the lifetime cost and QALY estimates. Secondly, the clinical trials did not report patients’ quality of life, the utility values used in this study were referenced from advanced GC patients in European populations, which might introduce potential estimation bias. Apart from this, the clinical trial data source exclusively involved Chinese populations, necessitating caution when extrapolating findings to other countries due to generalizability limitations. Furthermore, since the clinical trial only reported overall AEs incidence rates and the relatively short follow-up duration may have failed to capture immune-related long-term AEs, our model incorporated management costs and disutility only for SAEs during the first treatment cycle, without accounting for these potential long-term immune-related events.

## 5. Conclusion

This first-in-region economic evaluation concludes that sugemalimab plus CAPOX fails to demonstrate cost-effectiveness versus CAPOX monotherapy in first-line treating unresectable locally advanced/metastatic GC/GEJC regardless of PD-L1 expression levels under Chinese healthcare payer perspective. A price reduction for sugemalimab is required to achieve acceptable cost-effectiveness; however, the extent of price reduction needed would be relatively lower in regions with higher economic development levels.

## Supplementary Material

Supplementary Materials.docx

## Data Availability

The data that support the findings of this study are available from the corresponding author upon reasonable request (QX).
