# Supplementary material for: Cost-effectiveness of sugemalimab plus chemotherapy as first-line therapy in advanced gastric cancer and gastroesophageal junction cancer
Source: Ann Med. 2025 Dec 9;57(1):2583551. doi: 10.1080/07853890.2025.2583551 (PMC12697266; doi:10.1080/07853890.2025.2583551)
Supplement: Supplementary Materials.docx [file IANN_A_2583551_SM0107.docx]

***Supplementary Materials***

**Table S1.** CHEERS Checklist (2022)

| **Topic** | **No.** | **Item** | **Reported?** |
| --- | --- | --- | --- |
| **Title and abstract** |  |  |  |
| Title | 1 | Identify the study as an economic evaluation and specify the interventions being compared. | Yes |
| Abstract | 2 | Provide a structured summary that highlights context, key methods, results, and alternative analyses. | Yes |
| **Introduction** |  |  |  |
| Background and objectives | 3 | Give the context for the study, the study question, and its practical relevance for decision making in policy or practice. | Yes |
| **Methods** |  |  |  |
| Health economic analysis plan | 4 | Indicate whether a health economic analysis plan was developed and where available. | Yes |
| Study population | 5 | Describe characteristics of the study population (such as age range, demographics, socioeconomic, or clinical characteristics). | Yes |
| Setting and location | 6 | Provide relevant contextual information that may influence findings. | Yes |
| Comparators | 7 | Describe the interventions or strategies being compared and why chosen. | Yes |
| Perspective | 8 | State the perspective(s) adopted by the study and why chosen. | Yes |
| Time horizon | 9 | State the time horizon for the study and why appropriate. | Yes |
| Discount rate | 10 | Report the discount rate(s) and reason chosen. | Yes |
| Selection of outcomes | 11 | Describe what outcomes were used as the measure(s) of benefit(s) and harm(s). | Yes |
| Measurement of outcomes | 12 | Describe how outcomes used to capture benefit(s) and harm(s) were measured. | Yes |
| Valuation of outcomes | 13 | Describe the population and methods used to measure and value outcomes. | Yes |
| Measurement and valuation of resources and costs | 14 | Describe how costs were valued. | Yes |
| Currency, price date, and conversion | 15 | Report the dates of the estimated resource quantities and unit costs, plus the currency and year of conversion. | Yes |
| Rationale and description of model | 16 | If modelling is used, describe in detail and why used. Report if the model is publicly available and where it can be accessed. | Yes |
| Analytics and assumptions | 17 | Describe any methods for analysing or statistically transforming data, any extrapolation methods, and approaches for validating any model used. | Yes |
| Characterising heterogeneity | 18 | Describe any methods used for estimating how the results of the study vary for subgroups. | Yes |
| Characterising distributional effects | 19 | Describe how impacts are distributed across different individuals or adjustments made to reflect priority populations. | Yes |
| Characterising uncertainty | 20 | Describe methods to characterise any sources of uncertainty in the analysis. | Yes |
| Approach to engagement with patients and others affected by the study | 21 | Describe any approaches to engage patients or service recipients, the general public, communities, or stakeholders (such as clinicians or payers) in the design of the study. | Not applicable |
| **Results** |  |  |  |
| Study parameters | 22 | Report all analytic inputs (such as values, ranges, references) including uncertainty or distributional assumptions. | Yes |
| Summary of main results | 23 | Report the mean values for the main categories of costs and outcomes of interest and summarise them in the most appropriate overall measure. | Yes |
| Effect of uncertainty | 24 | Describe how uncertainty about analytic judgments, inputs, or projections affect findings. Report the effect of choice of discount rate and time horizon, if applicable. | Yes |
| Effect of engagement with patients and others affected by the study | 25 | Report on any difference patient/service recipient, general public, community, or stakeholder involvement made to the approach or findings of the study | Not applicable |
| **Discussion** |  |  |  |
| Study findings, limitations, generalisability, and current knowledge | 26 | Report key findings, limitations, ethical or equity considerations not captured, and how these could affect patients, policy, or practice. | Yes |
| Other relevant information |  |  |  |
| Source of funding | 27 | Describe how the study was funded and any role of the funder in the identification, design, conduct, and reporting of the analysis | Yes |
| Conflicts of interest | 28 | Report authors conflicts of interest according to journal or International Committee of Medical Journal Editors requirements. | Yes |

From: Husereau, D., Drummond, M., Augustovski, F., de Bekker-Grob, E., Briggs, A. H., Carswell, C., et al. (2022). Consolidated health economic evaluation reporting standards 2022 (CHEERS 2022) statement: Updated reporting guidance for health economic evaluations. *MDM Policy Pract.* 7(1)**,** 23814683211061097. doi:10.1177/23814683211061097

Table S2 Summary of the statistical goodness-of-fit of Kaplan Meier survival curves.

| Distribution | Sugemalimab, AIC | Sugemalimab, BIC | Placebo, AIC | Placebo, BIC |
| --- | --- | --- | --- | --- |
| **OS** | | | | |
| **PD-L1 CPS ≥ 5** | | | | |
| Exponential | 1315.134531 | 1318.619328 | 1357.531788 | 1361.004059 |
| Weibull | 1290.933904 | 1297.903498 | 1337.984323 | 1344.928865 |
| Gompertz | 1301.876005 | 1308.845599 | 1350.713017 | 1357.657559 |
| Log-normal | 1295.643162 | 1302.612755 | 1332.566168 | 1339.510709 |
| Log-logistic | 1287.41574 | 1294.385334 | 1330.362386 | 1337.306928 |
| **PD-L1 CPS ≥ 10** | | | | |
| Exponential | 698.7528071 | 701.6203415 | 724.7779583 | 727.6299885 |
| Weibull | 685.7730041 | 691.508073 | 720.4924182 | 726.1964787 |
| Gompertz | 690.5932977 | 696.3283666 | 724.4595686 | 730.1636292 |
| Log-normal | 686.3046463 | 692.0397152 | 720.4564717 | 726.1605322 |
| Log-logistic | 685.255978 | 690.9910469 | 718.9314939 | 724.6355544 |
| **PFS** | | | | |
| **PD-L1 CPS ≥ 5** | | | | |
| Exponential | 1199.981885 | 1203.466682 | 1213.482244 | 1216.954514 |
| Weibull | 1180.277767 | 1187.24736 | 1182.655268 | 1189.59981 |
| Gompertz | 1195.997413 | 1202.967007 | 1206.458169 | 1213.402711 |
| Log-normal | 1196.975035 | 1203.944629 | 1162.711219 | 1169.65576 |
| Log-logistic | 1167.343686 | 1174.31328 | 1164.53945 | 1171.483991 |
| **PD-L1 CPS ≥ 10** | | | | |
| Exponential | 630.7048498 | 633.5723842 | 629.4564281 | 632.3084583 |
| Weibull | 615.9907917 | 621.7258606 | 617.9911592 | 623.6952198 |
| Gompertz | 626.2473007 | 631.9823696 | 626.9084174 | 632.6124779 |
| Log-normal | 607.5278561 | 613.262925 | 609.9906255 | 615.694686 |
| Log-logistic | 609.2424745 | 614.9775434 | 613.7347457 | 619.4388063 |

Abbreviations: OS, Overall survival; PFS, Progression-free survival; CPS, Combined positive score; AIC, Akaike information criterion; BIC, Bayesian information criterion.

Table S3 (a) Parameter estimates for alternative parametric distributions

| Distribution | Parameter 1 | Parameter 2 | Sugemalimab | Placebo |
| --- | --- | --- | --- | --- |
| OS | | | | |
| Exponential | rate | - | 0.04489 | 0.05653 |
| Weibull | shape | scale | 1.466 / 20.697 | 1.3771 / 17.2543 |
| Gompertz | shape | rate | 0.04704 / 0.02784 | 0.03483 / 0.04094 |
| Log-normal | meanlog | sdlog | 2.7352 / 0.9721 | 2.5042 / 0.9545 |
| Log-logistic | shape | scale | 1.865 / 15.448 | 1.835 / 12.358 |
| PFS | | | | |
| Exponential | rate | - | 0.09041 | 0.12952 |
| Weibull | shape | scale | 1.3556 / 10.8941 | 1.4109 / 7.9682 |
| Gompertz | shape | rate | 0.03618 / 0.07233 | 0.0467 / 0.1017 |
| Log-normal | meanlog | sdlog | 2.0447 / 1.0097 | 1.7162 / 0.8155 |
| Log-logistic | shape | scale | 1.933 / 7.691 | 2.125 / 5.613 |

Table S3 (b) Sensitivity analyses with various parametric distributions

| Incremental Cost ($) | Incremental QALYs | ICER ($/QALY) |
| --- | --- | --- |
| Base case: Log-logistic OS; Log-logistic PFS | | |
| 44,455.46 | 0.22 | 206,024.50 |
| Log-normal OS; Log-normal PFS | | |
| 43,109.51 | 0.19 | 223,970.88 |
| Weibull OS; Weibull PFS | | |
| 45,060.10 | 0.17 | 271,534.71 |
| Gompertz OS; Gompertz PFS | | |
| 43,823.50 | 0.20 | 219,117.50 |
| Exponential OS; Exponential PFS | | |
| 46,978.08 | 0.23 | 202,817.00 |


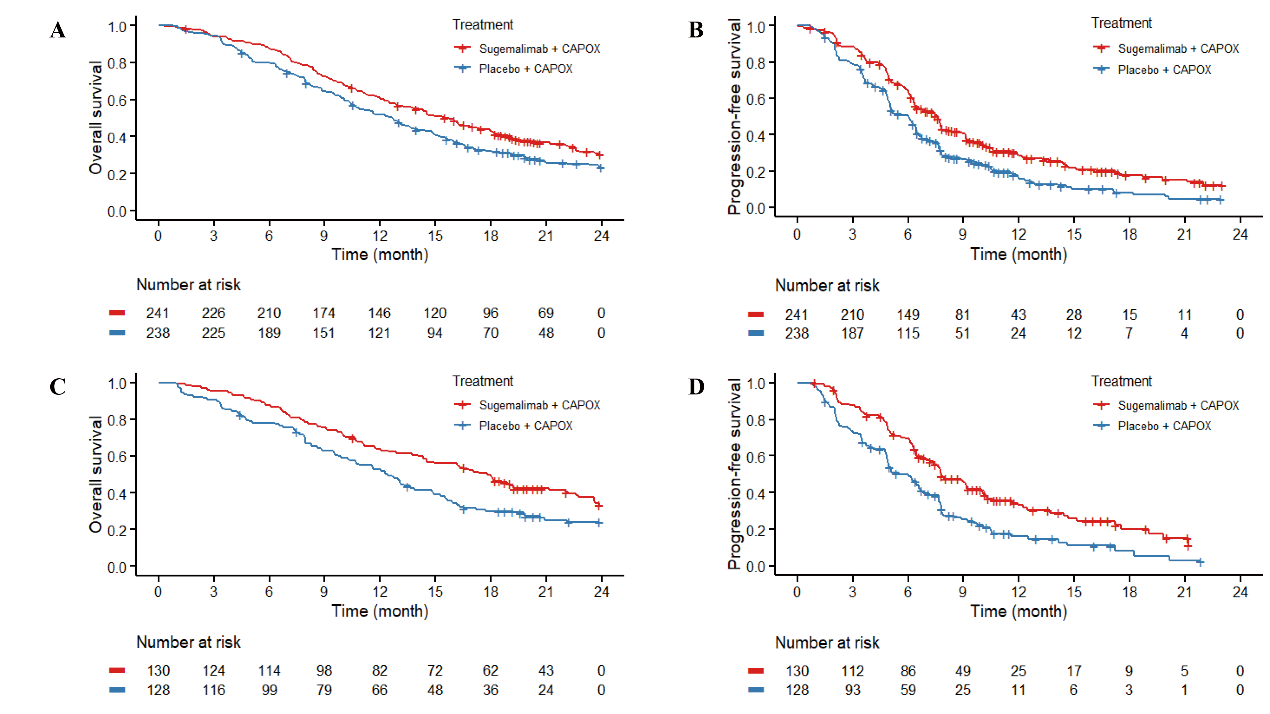


Figure S1 Reconstruction of Kaplan Meier survival curve. (A) OS curve in PD-L1 CPS≥5; (B) PFS curve in PD-L1 CPS≥5; (C) OS curve in PD-L1 CPS≥10; (D) PFS curve in PD-L1 CPS≥10. Abbreviations: CAPOX, capecitabine and oxaliplatin;


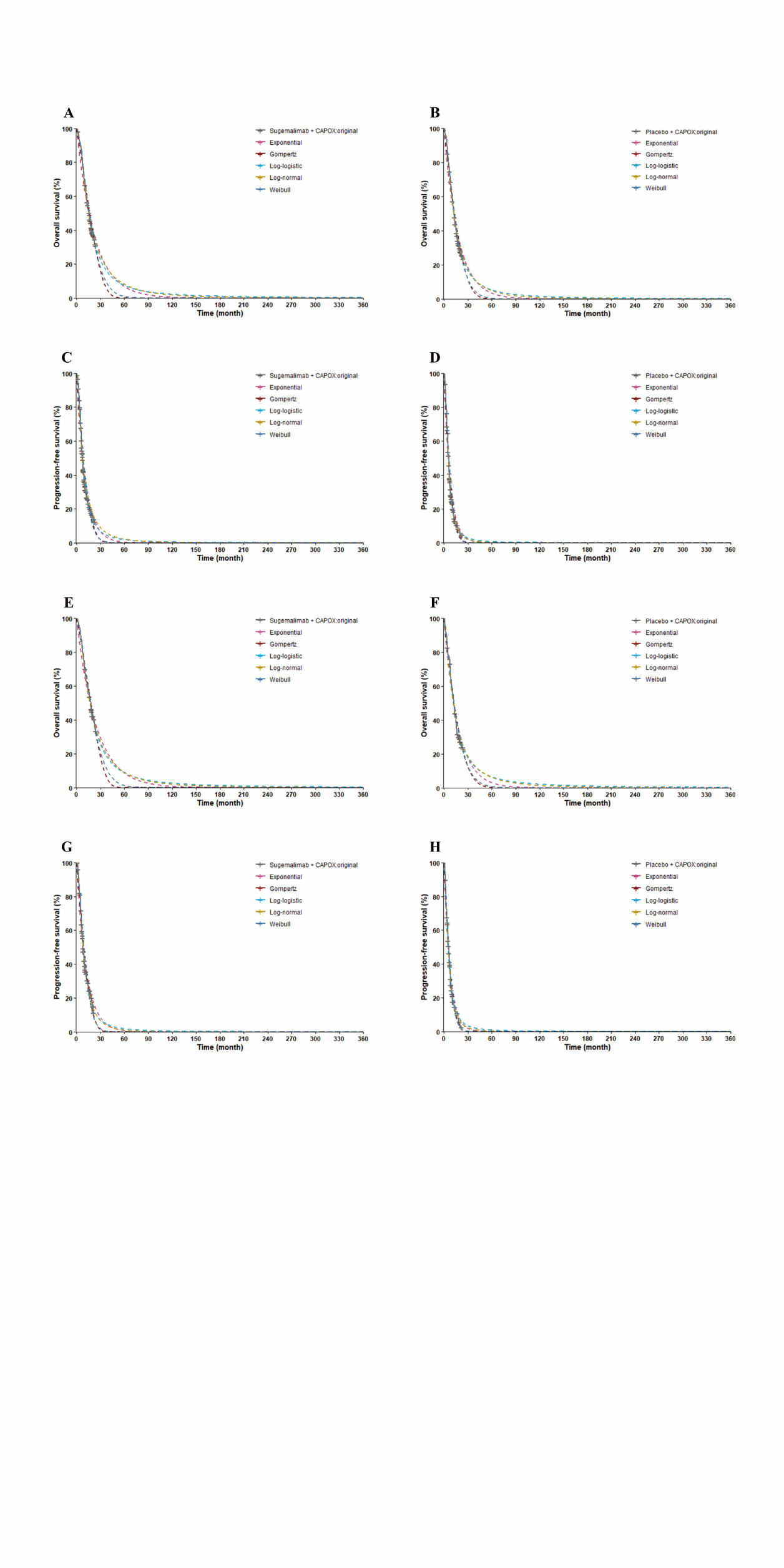


Figure S2 Fitting and extrapolation of Kaplan Meier survival curve. (A)The results of sugemalimab plus COPAX OS curve in PD-L1 CPS≥5 group; (B)The results of placebo plus CAPOX OS curve in PD-L1 CPS≥5 group; (C)The results of sugemalimab plus CAPOX PFS curve in PD-L1 CPS≥5 group; (D)The results of placebo plus CAPOX PFS curve in PD-L1 CPS≥5 group; (E)The results of sugemalimab plus CAPOX OS curve in PD-L1 CPS≥10 subgroup; (F)The results of placebo plus CAPOX OS curve in PD-L1 CPS≥10 subgroup; (G)The results of sugemalimab plus CAPOX PFS curve in PD-L1 CPS≥10 subgroup; (H)The results of placebo plus CAPOX PFS curve in PD-L1 CPS≥10 subgroup. Abbreviations: CAPOX, capecitabine and oxaliplatin;
